# Outcomes of olecranon fractures in adolescents: comparison of tension band wiring and Herbert screw fixations

**DOI:** 10.3389/fped.2023.1269628

**Published:** 2024-01-10

**Authors:** Weiwei Yang, Xintao Zhang, Dong Sun, Shaobin Jin, Junfei Chen, Yang Li

**Affiliations:** Department of Pediatric Surgery, Qilu Hospital of Shandong University, Shandong, China

**Keywords:** children, olecranon fracture, Herbert screw fixation, epiphyseal growth plate, tension band wiring

## Abstract

**Purpose:**

Olecranon fracture is considered intra-articular when there is obvious displacement or an irregular articular surface. Such fractures should be reduced and fixed via surgery. No clear indications regarding the surgical technique to be adopted exist. Therefore, this study aimed to compare the outcomes of tension band wiring (TBW) and Herbert screw fixations for olecranon fractures.

**Methods:**

We retrospectively analyzed the clinical data of 29 children with olecranon fractures. They were divided into the tension band wiring and Herbert screw groups. We assessed early epiphyseal closure and maximum length of the ulna using radiography. Patients were clinically evaluated using the average QuickDASH score.

**Results:**

Both groups had good radiological outcomes. Herbert screws demonstrated advantages in terms of bleeding, operative time, intraoperative blood loss, surgery duration, and particularly the QuickDASH score (1.57 vs. 4.18, *p* < 0.05). Complications, including needle loosening and bursitis, occurred in five cases in the TBW group. Six cases had premature physis plate closure, and no difference was observed in limb length at 6 months after surgery.

**Conclusion:**

Compared with TBW, Hebert screws demonstrated better clinical outcomes and lesser postoperative complications in the treatment of ulnar olecranon fractures in children. However, long-term follow-up is required to assess the effects of screws on the ulnar physis plate and ulna length.

## 1 Introduction

Olecranon fracture is considered intra-articular when the fracture has an apparent displacement or unsmooth articular surface and should be reduced and fixed via surgery to restore the normal para-position and to reduce elbow joint dysfunction, such as elbow stiffness, malunion, and traumatic arthritis. Olecranon fractures account for approximately 4%–7% of elbow fractures in children (1). Approximately 20% of cases are accompanied by other injuries to the ipsilateral elbow joint (2).

However, surgical indications remain controversial. It is generally believed that when the fracture block separation distance is <2 mm or 2–4 mm, with an elbow flexion of 90°, the fracture is stable, the joint surface is smooth, and the active anti-gravity extension of the elbow and the fracture is undisplaced, conservative treatment can be chosen. However, when the articular surface is unsmooth or the displacement is >4 mm, it could be an indication for open reduction and internal fixation (3, 4).

Surgical internal fixation techniques include tension-band wiring (TBW), tension-band suturing (TBS), surgery using screws, and other approaches,and Some scholars tried to use bioabsorbable compression screws or polyethylene tension band for fixation of displaced olecranon fractures (5). However, open reduction and TBW are the “gold standard” for treating olecranon fractures (6). The tension band suture can convert the tension through the fracture site's posterior cortex into pressure on the articular surface to increase the fracture fixation's stability and to prevent displacement. Its main disadvantages include steel needle displacement and steel wire stimulation of the skin. Additionally, a second incision is needed for removing the fixation (7). Screw fixation for olecranon fractures can provide sufficient pressure with minimal trauma. Currently, it is mainly used in children with osteogenic insufficiency (9). Due to relative osteoporosis in children with imperfect osteogenesis, the adhesion between the screw and bone is insufficient, leading to graft loosening. However, in relevant studies, no significant difference was observed between screw and tension-band fixation in adults. Corradin et al. reported good results with TBW and screw fixation for olecranon fractures in healthy children (9). Another concern regarding screw fixation is its effect on the epiphyseal plates. Currently, evidence to compare the efficacy of TBW and compression screw fixation for isolated olecranon fractures in healthy children is insufficient. The effect of screw fixation on the ulnar olecranon epiphyseal plate in children has rarely been investigated.

This study aimed to compare the clinical outcomes of open reduction with TBW fixation and closed reduction with Herbert screw internal fixation for treating olecranon fractures in children and to explore the possible effects of internal fixation device on olecranon epiphysis and ulna growth in children with olecranon fractures.

## 2 Materials and methods

Children (*n* = 29) with ulnar olecranon fractures who underwent surgery between January 2017 and June 2022 in our treatment center, Qilu Hospital of Shandong University, were included in the study. There were 18 boys and 11 girls, with 11 cases of fractures on the left side and 18 on the right. The causes of injury included 24 cases of falls and 5 of traffic accidents. The age range was 9–14 years. According to the different internal grafts, the children were divided into the TBW (group A: 12 patients) and Herbert screw (group B: 17 patients) groups. The average age was 11.46 (11.6 ± 1.20 vs. 11.3 ± 1.33) years. Data on the sex, age, and fracture side of both groups were statistically analyzed, and the difference was not significant (*p* > 0.05, Table 1). All the cases were of recent fractures, and the period from injury to surgery was 1–5 (average period 2.42 ± 1.24 vs. 2.24 ± 0.76) days. The Medical Ethics Committee of Qilu Hospital of Shandong University approved this study (approval number KYLL-2020008-165).

**Table 1:** Characteristics of the patients.

Number	Age (year)	Side (L:R)	Sex (M:F)	Operation waiting time (day)	Surgery duration (min)	Amount of bleeding	Follow-up	QuickDASH score
12	11.6	5:7	7:5	2.4	82.5	39.1	12.2	4.78
17	11.3	6:11	11:6	2.2	32.1	4.9	13.5	1.57

### 2.1 Inclusion and exclusion criteria

The inclusion criteria were as follows: isolated closed ulnar olecranon fractures or fractures at other sites of the forearm that did not require surgery; fracture displacement of 2–4 mm with an unsmooth joint surface or a displacement of >4 mm; patients who underwent open reduction TBW or closed reduction screw fixation; age 9–14 years; and follow-up of not less than 6 months.

The exclusion criteria were ulnar olecranon fractures combined with fractures of other sites of the forearm, which require surgery; open fracture of the ulnar olecranon, pathological fracture, or comminuted fracture; and age <9 years or >14 years. The olecranon epiphyseal plate on the radiograph was closed.

### 2.2 Surgical methods

Experienced pediatric orthopedic surgeons performed both procedures, The choice of internal fixation depends was based on the physician's discretion and guardian's preference. After inducing anesthesia, the patient was placed supine, and the affected limb was placed on a C-arm and disinfected.

#### 2.2.1 TBW group

**Figure 1:**
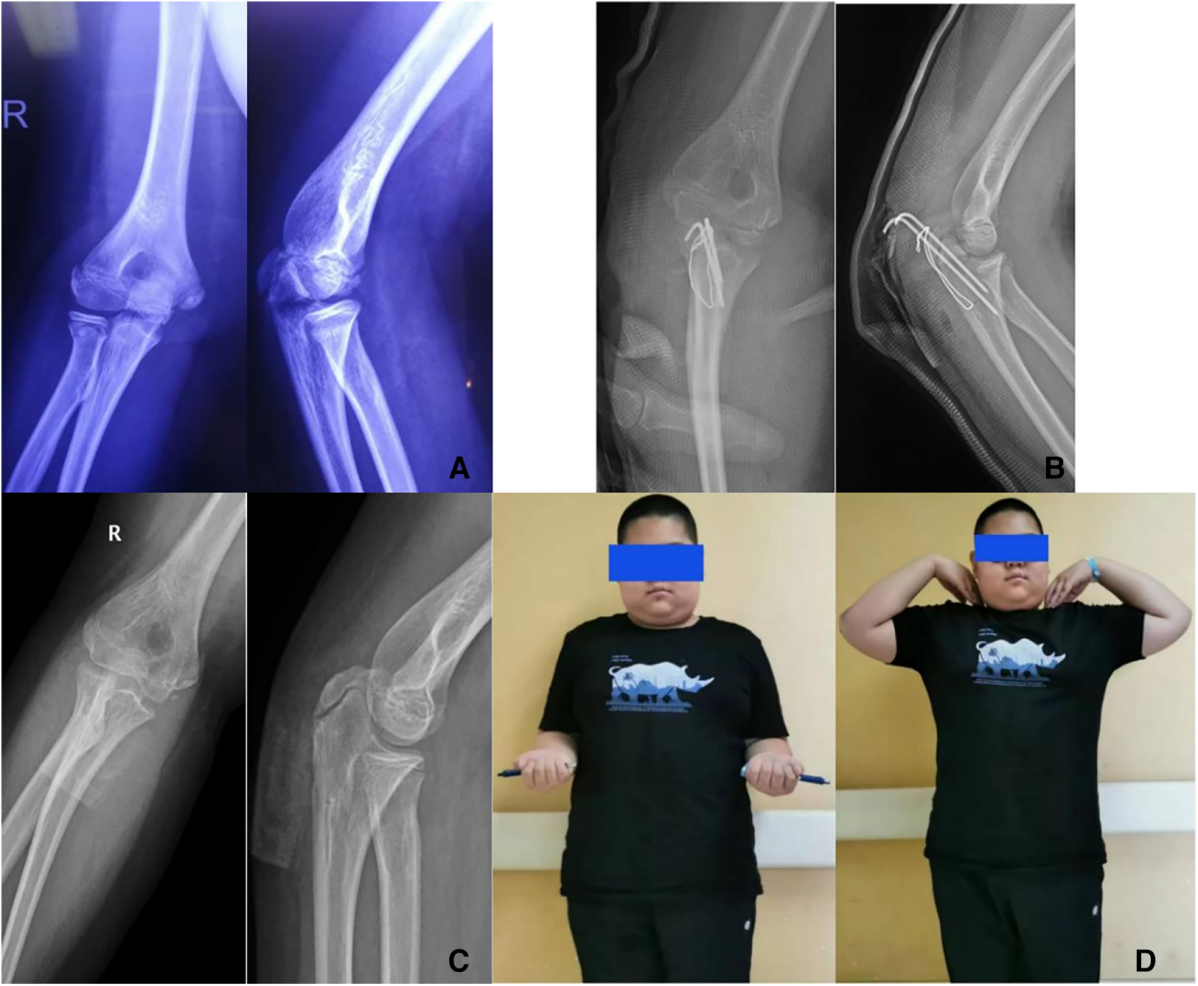
(**A**) Preoperative images; Anteroposterior (AP) and lateral radiographs in a 12-year-old boy with an olecranon fracture. (**B**) Postoperative images from the 1-day follow-up and AP and lateral radiographs in the same patient treated with open reduction and TBW fixation. (**C**) Follow-up images obtained at 6 months after surgery in the same patient. (**D**) Functional outcomes in the same patient at 6 months after surgery. Before removing the TBW screw, we recorded the patient's elbow range of motion in flexion, extension, pronation, and supination, forearm length, and QuickDASH score.

A longitudinal posterior incision of approximately 6 cm was made in the proximal ulna. The fracture was exposed, the towel clamp was temporarily reduced, and the fractured piece was immobilized. Based on the child's age, two smooth Kirschner needles with a diameter of 1.6 mm or 2.0 mm were selected. Two parallel Kirschner wires were longitudinally passed from the tip of the olecranon into the distal part of the ulna and close to the articular surface in front of the ulnar olecranon, and the distance between the Kirschner needles was approximately 0.8 cm. The distal point of the Kirschner needle was inserted into the cortex during the distal coronal process. We drilled holes on both sides of the ulnar crest, approximately 4 cm from the distal end of the fracture line and perpendicular to the longitudinal axis of the ulna. We selected a steel wire with an appropriate diameter to pass through the bone tunnel, cross-fixed in an “8” figure near the fracture line. We tightened and knotted the wires around the Kirschner needle tail, bent and cut the needle tail, and sutured the incision (Figure 1).

#### 2.2.2 Herbert screw group

**Figure 2:**
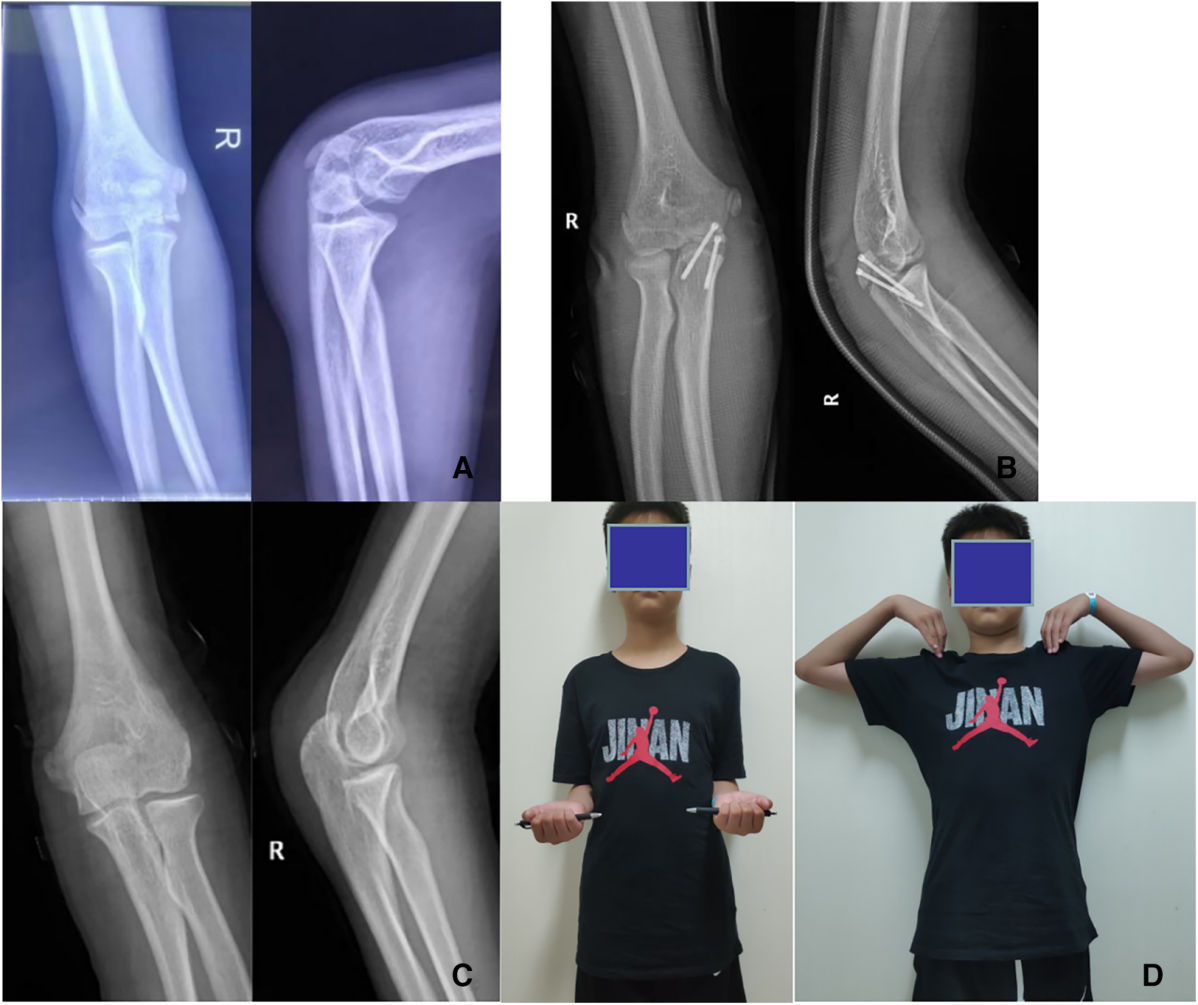
(**A**) preoperative images; AP and lateral radiographs in a 13-year-old boy with an olecranon fracture (**B**) postoperative images from the 1-day follow-up and AP and lateral radiographs in the same patient treated with closed reduction and Herbert screw fixation. (**C**) Follow-up images obtained at 6 months after surgery in the same patient. (**D**) Functional outcomes in the same patient at 6 months after surgery. Before removing the cannulated screw, we recorded the patient's elbow range of motion in flexion, extension, pronation, and supination, forearm length, and QuickDASH score.

The elbows were extended to reduce the traction of the triceps muscles on the fracture. Sometimes temporarily inserting a Kirschner needle proximal to the fracture and using the joystick technique to assist the reduction was required. After fracture reduction, two 0.8-mm Kirschner wires were inserted as guide needles from the proximal to distal points to fix the fractures temporarily. Two Herbert screws with a 3.0-mm diameter were implanted in the direction of the guide needle, which were then removed (Figure 2).

### 2.3 Postoperative treatment and evaluation

Patients in both groups were immobilized using a plaster cast postoperatively. AP and lateral radiographs of the elbow joint were reviewed on the first day, 2 weeks, and 4–6 weeks after surgery. Elbow joint function exercises were gradually performed after removing the cast after 4–6 weeks based on the fracture healing. At the 6-month follow-up, we used QuickDASH, bilateral anteroposterior (AP) and lateral radiographs to assess elbow joint function, elbow flexion and extension range, maximum length of ulna, and epiphysis closure. Patients were asked if they wanted their internal fixation removed. All patients had their internal fixation removed at 6–9 months after surgery.

#### 2.3.1 TBW group

The patients in this group underwent a procedure through the original incision to expose the TBW, which was then removed.

#### 2.3.2 Herbert screw group

The patient was placed in the supine position, and the affected limb was placed on a C-arm x-ray device and sterilized. A 0.8-mm Kirschner wire was inserted into the hollow screw and confirmed on AP and lateral radiographs. A 0.5-cm incision was made in the skin around the Kirschner wire, and the Herbert screw was unscrewed in the direction of the Kirschner wire. Compared to TBW, the use of a hollow screw resulted in a smaller incision and less surgical trauma.

### 2.4 Statistical methods

IBM SPSS Statistics for Windows, version 19.0 (IBM Corp., Armonk, NY, USA) was used for the analysis and comparison. The data were represented using means ± standard deviations. The data of both groups were compared using the t-test for two independent samples or Mann–Whitney *U* test. The threshold for statistical significance was *p* < 0.05.

## 3 Results

All 29 children were followed-up for 9–22 months, averaging 12.9 months. There were 12 patients in the TBW group (average follow-up period of 12.2 months) and 17 in the Herbert screw group (average follow-up period of 13.5 months). The average operation time of the Herbert screw group was 32.1 min less than that of the TBW group 82.5 min (*P* < 0.01) The volume of blood lost in the Herbert screw group was 4.9 ml, which was lower than that in the TBW group 39.0 ml (*P* < 0.01) (Table 1).

The mean QuickDASH scores in the TBW and Herbert screw groups were 4.78 and 1.57, respectively. In the TBW group, the steel needles loosened and were therefore withdrawn in three patients; further, two developed bursitis at the proximal ulna, which caused pain. Nine of the 12 patients in the TBW group strongly desired to have their internal fixation removed, especially those with a low body mass index. Many patients had difficulty tolerating foreign bodies and tissue scarring behind the elbow (Tables 2, 3).

**Table 2:** Group of patients treated by open reduction and TBW fixation.

Patient	Age at trauma	Sex	Side	Operation waiting time (day)	Surgery duration (min)	Amount of bleeding (ml)	Follow-up	Quick DASH	Aspiration of removing internal fixation
1	11.3	M	L	1	80	40	11	3.33	Y
2	10.2	F	R	2	90	30	13	5	N
3	13.6	M	R	4	70	30	9	5	Y
4	10.9	M	R	3	110	40	9	4.14	Y
5	13.7	F	R	5	80	20	15	6.67	Y
6	12.4	M	L	2	90	50	13	2.5	N
7	10.0	M	L	3	80	40	12	8.33	Y
8	10.8	F	R	1	100	40	11	4.14	Y
9	10.8	M	L	1	70	50	14	3.33	Y
10	11.7	F	R	3	80	40	10	4.14	Y
11	12.2	M	L	2	70	50	14	5	Y
12	11.7	F	R	2	70	40	15	5.83	N

**Table 3:** Group of patients treated by closed reduction and Herbert screw fixation.

Patient	Age at trauma	Sex	Side	Operation waiting time	Surgery duration (min)	Amount of bleeding (ml)	Follow-up	Quick DASH	Aspiration of removing internal fixation
1	10.0	M	R	2	40	5	13	1.67	N
2	13.2	M	L	2	25	5	15	0	N
3	12.1	F	R	2	30	3	13	3.33	Y
4	9.8	M	R	2	30	5	12	2.5	N
5	11.2	F	L	3	25	8	14	1.67	N
6	10.6	M	R	4	40	5	22	0	N
7	12.5	M	R	3	30	5	15	0	N
8	12.3	F	R	3	30	2	14	0.83	Y
9	9.8	M	L	2	40	5	15	3.33	N
10	9.6	M	L	2	30	5	10	1.67	N
11	13.0	F	R	1	30	3	9	1.67	N
12	10.8	F	R	3	25	4	13	0.83	N
13	11.7	M	R	2	30	5	14	2.5	N
14	10.5	M	L	2	25	5	14	3.33	N
15	10.6	M	L	2	50	5	15	1.67	N
16	13.9	M	R	1	30	8	9	1.67	N
17	11.3	F	R	2	35	5	12	0	N

No patient in the Herbert screw group had foreign body irritation, implant migration, or osteoarthritis. Two of the 17 patients in this group strongly desired to have their internal fixation removed. The internal fixation was removed in most patients at 6–8 months after surgery because cannulated compression screws might affect the growth of epiphyseal or secondary ossification centers. Before removing the internal fixation, elbow function was assessed in both groups, and the elbow flexion of children in the Herbert screw group was better than that of those in the TBW group. No significant difference was observed in elbow flexion and forearm pronation or supination between both groups. In the sixth month, we assessed the limb length of affected sides in two group patients, and no difference in limb length existed in both groups (Table 4).

**Table 4:** Comparison of ROM and ulna length outcomes between the TBW and Herbert screw fixation.

	TBW	Herbert screw fixation	*P*
Elbow flexion (°)	135 (135, 140)	140 (140, 145)	0.024
Elbow extension (°)	0 (0, 5)	0 (0, 5)	0.586
Forearm pronation (°)	85 (80, 85)	85 (85, 85)	0.245
Forearm supination (°)	85 (81.25, 90)	85 (85, 90)	0.499
Maximum length of the ulna (cm)	22.72 ± 1.22	22.46 ± 1.37	0.614

## 4 Discussion

Ulnar olecranon fractures account for 4%–7% of all elbow fractures in children. Because of the effective fixation methods and excellent results (3), TBW has been regarded as the gold standard for olecranon fractures. However, hardware irritation, persistent joint pain, scar hyperplasia and other common complications trouble the patients. Although compression screw fixation for ulna olecranon fractures has been well described in adults (10), reports in the pediatric population are very few. This study investigated the effects of different fixation modalities on ulnar olecranon fractures.

Ulnar olecranon fracture treatment using TBW technology is a relatively mature surgical method, TBW is considered the gold standard for the surgical treatment of ulnar olecranon fractures in children (11). However, it requires a relatively long incision and adequate exposure of the proximal ulnar olecranon, often causing postoperative incision pain in children. Longer surgical incisions can also cause cosmetic challenges (11). In addition, owing to the low subcutaneous fat in the olecranon area and high incision tension, the needle tip can easily penetrate the skin, infecting the incision (12). Furthermore, this technology can loosen or displace the Kirschner needle, break the steel wire, and cause needle tail irritation of the skin and soft tissues, causing pain (13). In our study, grafts loosened in three cases, causing the tips of the Kirschner needle to touch the skin surface without fracture displacement or local infection during subsequent follow-up. In addition, two children developed bursitis at the proximal ulna, which caused pain and decreased the range of motion (ROM) of the elbow joint. Studies have revealed that the hardware removal rate is significantly higher in children (63%) than in adults (6%–25%), possibly due to elbow dysfunction caused by Kirschner needle stimulation or the parents' willingness. In the TBW group, removal was performed under general anesthesia using an open incision almost identical to the original incision.

Good reduction and stable fixation are critical for recovery from olecranon fractures. This differs from that observed among the children in the TBW group, as no child in the Herbert screw group experienced graft loosening or local soft tissue irritation. When the Herbert screw was used to fix the ulna olecranon fracture, the ends of the screws were embedded in the ulna's periosteum, and foreign body rejection rarely occurred. In addition, soft tissue stimulation by the screw during elbow movement was negligible. Previous studies have suggested that screw fixation might result in graft loosening in patients with ulnar olecranon fractures with OI, possibly due to insufficient internal fixation and bone adhesion in children with osteoporosis (8). In this group, no patient experienced internal fixation loosening. However, the hollow screw did not reach the contralateral cortex in some patients. We believe that the hollow screw can provide sufficient pressure to the olecranon. During fixation, the screws are pressurized by the head and tail thread difference and number of screw-in threads to reduce the fracture gap and to achieve a better reduction. Hollow compression screws to fix ulnar olecranon fractures can provide sufficient pressure early to reset the fracture. Within 12 h of fixation, 39%–55% of compression disappears; however, good initial compression remains important (14). In addition, the fixation angle of the patients was maintained at approximately 45° of elbow flexion, which helped alleviate the triceps tension, indicating that the Herbert screw could provide sufficient holding force to resist the triceps pull.

All cases in the compression screw group achieved closed reduction, and Kirschner and guide needles were used to fix the fracture fragment temporarily. Compression screws were inserted along the guide needles, and the fracture gap gradually closed. In this procedure, a mini incision of approximately 0.5 cm is required. The compression screw group had apparent advantages in operation time, volume of blood loss, and postoperative pain compared with the TBW group. This is mainly due to the minimally invasive nature of the operation and sufficient stability of the screws. Additionally, when removing the compression screw, inserting the Kirschner needle along the original surgical incision and removing the screw along the guide needle to achieve a minimally invasive operation are necessary.

Hollow screw fixation is often controversial in children with ulnar olecranon fractures because of potential damage to the growth plate caused by a large screw. Compression screw fixation for ulnar olecranon fractures is mostly used in adults and children with osteogenesis imperfecta (15, 16), and the main concern is an injury to the ulnar olecranon epiphyseal plate in children, leading to forearm deformity and growth arrest (17). Ulnar olecranon epiphyseal ossification centers appear at 9–11 years of age and begin to heal with the ulnar shaft around the age of 17 years, whereas 15% of the ulna growth depends on the proximal growth plate, and the growth rate drops to 5% by the age of 8 years (18). In recent years, some researchers have applied screws to ulnar olecranon fractures in healthy children (9, 19) and achieved a clinical efficacy similar to that of tension band wires. Bilateral AP and lateral radiographs were examined after 6 months to study the effect of screws on premature epiphyseal closure and extremity length. Despite the premature closure of epiphyseal plate in six cases, no angle and ulnar length deformity was observed and no significant difference was observed in the elbow ROM score. Considering the smaller growth potential of the remaining olecranon in older children and stimulation of the epiphysis by the screw's small diameter and minimally invasive approach to preserve the olecranon blood supply, we suggest that the use of hollow screws results in negligible ulnar growth in older children. However, long-term follow-up is required to confirm whether the ulna is affected after the epiphyseal plate is completely closed.

We used QuickDASH and elbow ROM as evaluation index at 6 months after surgery. This scoring system can balance children's subjective feelings with the doctor's objective evaluation (20). In the TBW group, more than one child had a low score owing to a restricted ROM and pain. However, in the Herbert Screw group, children had higher acceptance, lower QuickDASH scores, and lesser endoplant-induced pain and activity restriction than those in the TBW group. We investigated the subjective feelings of the children and their parents when they had a strong desire to remove the internal fixation. Compared with those in the hollow screw group, patients in the TBW group strongly desired to have their internal fixation removed. Subjective factors are involved in this process; however, the compression screw group can be better accepted in evaluating the result.

This study had some limitations. First, this was a single-center study, and the sample size was small. Second, this study was retrospective, and the choice of surgical modality depended on the preference of the surgeon rather than randomization. Third, the follow-up duration was short. Finally, no data were provided on the ulna evaluation when the ulnar epiphysis was closed.

In conclusion, according to our study, TBW and screws achieved similar clinical results in treating ulnar olecranon fractures in children. Screw fixation is not contraindicated; rather, it offers unique advantages in terms of bleeding, operative time, and postoperative complications. Long-term follow-up is needed to observe ulnar growth after screw fixation.

## Data Availability

The original contributions presented in the study are included in the article/Supplementary Materials, further inquiries can be directed to the corresponding author.
