# Corrigendum: Outcomes of olecranon fractures in adolescents: comparison of tension band wiring and Herbert screw fixations

**DOI:** 10.3389/fped.2024.1484562

**Published:** 2024-09-23

**Authors:** Weiwei Yang, Xintao Zhang, Dong Sun, Shaobin Jin, Junfei Chen, Yang Li

**Affiliations:** Qilu Hospital, Shandong University, Jinan, Shandong, China

**Keywords:** children, olecranon fracture, Herbert screw fixation, epiphyseal growth plate, tension band wiring

A Corrigendum on Outcomes of olecranon fractures in adolescents: comparison of tension band wiring and Herbert screw fixations By Yang W, Zhang X, Sun D, Jin S, Chen J and Li Y (2024). Front. Pediatr. 11:1269628. doi: 10.3389/fped.2023.1269628

In the published article, there was an error in the legend for **Figure 1** as published. The part labels (B) “Postoperative images from the 1-day follow-up and AP and lateral radiographs in the same patient treated with closed reduction and Herbert screw fixation” and (D) “Functional outcomes in the same patient at 6 months after surgery. Before removing the cannulated screw, we recorded the patient's elbow range of motion in flexion, extension, pronation, and supination, forearm length, and QuickDASH score”, were incorrect. The corrected legend appears below.

**Figure 1 (A)** preoperative images; anteroposterior (AP) and lateral radiographs in a 12-year-old boy with an olecranon fracture. **(B)** Postoperative images from the 1-day follow-up and AP and lateral radiographs in the same patient treated with open reduction and TBW fixation. **(C)** Follow-up images obtained at 6 months after surgery in the same patient. **(D)** Functional outcomes in the same patient at 6 months after surgery. Before removing the TBW screw, we recorded the patient's elbow range of motion in flexion, extension, pronation, and supination, forearm length, and QuickDASH score.

The authors apologize for this error and state that this does not change the scientific conclusions of the article in any way. The original article has been updated.

